# Factors of stability and sustainable development in Jordan in its first centenary 1921–2021 (an analytical descriptive study)

**DOI:** 10.1016/j.heliyon.2023.e20993

**Published:** 2023-10-18

**Authors:** Mohammad Saleh Bani Issa

**Affiliations:** Department of Political Science, School of Law, Jadara University, Jordan

**Keywords:** Jordan, Stability, Sustainable, Regime, Legitimacy, National unity, Belonging and loyalty

## Abstract

The study aims to discover the variables that have contributed to the stability of Jordan's political system, despite the barriers and conditions that the Jordanian state has faced for more than a century in the world's tensest region. It began with a hypothesis: there is a link between Jordan's stability and the legitimacy of the regime, which is founded on voluntary consent between the leadership and the people, as well as loyalty to the leadership, belonging to the homeland, the rule of law, and respect for the constitution. The study represented Jordan's stability features, stages of development, and current position on the world's political map in the first century. The study's scientific and practical significance lies in clarifying Jordan's nature and the growth, progress, and prosperity that this country is experiencing at all levels, despite the challenges it faced during the centennial; and in assisting researchers, scholars, politicians, and decision-makers in similar countries to Jordan in determining how to benefit from Jordan's experience as a role model. The study used historical, descriptive-analytical, systems analytical, and decision-making methodologies to answer the study's questions and examine its premise. It concluded that the ruling regime's religious, historical, and political legitimacy, as well as its relationship with the Hashemites, the nature of Jordanian society, as well as the rule of law and institutions, national unity, and the strong mutual relationship between the leadership and the people, all contributed to Jordan's century-long process of political stability and development.

## 1 Introduction

To begin with, Jordan's history dates back to antiquity; the Jordanian territory witnessed the stages of life's growth and numerous civilizations, leading to the foundation of the modern state and its birth a century ago. Jordan's archaeological sites attest to the antiquity of life in the region, as well as the periods and civilizations that Jordan has witnessed throughout history. The Copper Age and the Bronze Age are two of these antiquities. The archaeological sites in Jordan are representative of the history of the Edomite Semitic State, the Moabite Semitic State, and the Ammonite Semitic State. The Nabataean empire was created on Jordanian soil during the Iron Ages, and the Nabateans were pure Arabs who took control of southern Jordan, established one of the region's most important civilizations, and made Petra their capital. This period lasted from 500 BCE to 106 AD. Jordan was occupied by Persian (539–333 BCE), Greek (332–64 BCE), Roman (64 BCE to 395 AD), and Byzantine (395–636 AD) forces throughout history [1]. Jordan became a portal to the conquest of the Levant during the Islamic era, through which Islamic forces traveled to spread Islam, and witnessed the Battle of Muta and the Battle of Yarmouk. Several civilizations succeeded in Jordan after the Islamic conquest, beginning with the Umayyad, Abbasid, and Fatimid civilizations until 1099 AD, when Jordan fell under Frankish occupation until Salah al-Din al-Ayyubi came and expelled the Franks from Jordan and defeated them in the Battle of Hattin in 1187. The Ayyubids remained in Jordan until the commencement of the Mamluk kingdom in 1250 until 1516, when the Ottomans defeated the Mamluks and the Levant, including Jordan, remained under Ottoman control until the end of 1918 [2].

Jordan became part of the Hashemite Arab Kingdom of Syria after the First World War ended in 1918, under the leadership of King Faisal bin al-Hussein. Following the Battle of Maysalun on July 24, 1920, Jordan experienced a period of governmental vacuum until August 21, 1920, which resulted in the intensification of unrest and disruption of the region's conditions at all levels. On August 21, 1920, after meeting with various Jordanian leaders, Britain decided to construct a number of Jordanian local administration councils. Local administrations councils existed from August 21, 1920, to March 21, 1921, when Prince Abdullah ibn Al Hussein arrived and formed the Emirate of Transjordan on April 11, 1921 [3].

It is worth emphasizing that Jordan and the Hashemite Kingdom of Jordan are the names of the Jordanian land; the term Jordan is derived from the eternal Jordan River. It is a 360-km-long river that runs from Mount Hermon to the Dead Sea. The Jordan River was named after the confluence of one of its tributaries, JOR (Al-Hasbani), and another, DAN (Banias), which formed the Jordan River [4]. As a result, the Jordanian state got its name from here. Jordan is officially known as the Hashemite Kingdom of Jordan. It is critical to understand the Jordanian state's triple name; the term “kingdom” refers to the fact that the Jordanian state's ruling regime is a monarchy, while the term “Jordanian” refers to the Jordan River, as mentioned above, and the term Hashemite refers to Hashem, the Prophet Muhammad's great-grandfather, and from him descends the lineage of the Jordanian state's royal Hashemite family. Jordan's King Abdullah II Ibn al-Hussein is the forty-third generation connected to the Prophet Muhammad, peace and blessings be upon him [5].

## 2 Study's general framework

It is crucial to analyze the factors that have contributed to the political stability of Jordan's political system. Additionally, it is important to highlight the stages of development and accomplishments that Jordan has achieved over the past century, despite the challenges and difficult conditions it has faced in a region known for its high levels of tension since the beginning of the last century until the present day. The study commences by proposing the hypothesis that a correlation exists between the stability of Jordan and the legitimacy of its regime. This legitimacy is founded upon voluntary consent between the leadership and the populace, loyalty towards the leadership and a sense of belonging to the homeland, adherence to the rule of law, and respect for the constitution.

The primary objectives of the study are to address the following inquiries: What are the factors that contribute to the political stability of Jordan, what are the different stages of political development that Jordan has undergone, and how does Jordan's position on the world political map compare regionally and internationally during the first century of its modern era. The questions formulated in this study aim to accomplish the following objectives: identify the factors contributing to Jordan's political stability, delineate the stages of political development in Jordan, and ascertain Jordan's position on the global political landscape, both regionally and internationally, during the first centenary of its modern era.

It is of utmost importance to showcase the true essence of Jordan to the world, highlighting its growth, progress, and prosperity across various domains. Despite the challenges faced over the past century, this study aims to shed light on the remarkable resilience of the country. Emphasizing the scientific and practical significance of this research is crucial at this juncture. From a scientific standpoint, this study provides an opportunity for researchers, scholars, and students to gain a deeper understanding of Jordan and apply their newfound knowledge in their future studies and research endeavors. Furthermore, the practical value of this study extends to lawmakers and decision-makers in countries similar to Jordan, who can draw insights from Jordan's experiences as a model for their own governance and development strategies.

## 3 Literature review and materials

The study covers three axes: the first axis analyzes the factors of political stability in Jordan; the second axis discusses the stages of political development in Jordan; and the third axis discusses and investigates Jordan's place on the world's political map, regionally and internationally, in the first centenary of its modern era [6].

### 3.1 The first axis: Jordanian political stability factors

The legitimacy of the ruling regime, the character of Jordanian society, and the rule of law and institutions are the three stabilizing factors that have played a significant role in establishing security and stability in Jordan from the founding of the Jordanian state to the present. The three stabilizing factors are discussed in the order listed below: [7].

**First Factor**: the legitimacy of the ruling regime:

Jordan's ruling system is religiously, historically, and politically legitimate. Accordingly, the study evaluates the Hashemites' religious, historical, and political legitimacy as follows [8].

- *Religious legitimacy and its relationship to the Hashemites:* Religious legitimacy is tied to the Hashemite family's relationship with the Prophet Muhammad (peace and blessings be upon him). The Hashemite family can trace its roots back to Hashem, a member of the Quraish tribe. Hashem is the eldest grandson of Mecca's King Qusay bin Kilab; Qusay bin Kilab is the Prophet Muhammad's great-grandfather. And there are eight generations between Quraish and Hashim. Abdul Muttalib was born to Hashem, and Abdul Muttalib fathered four children, including Abdullah and Abu Talib. Abdullah was the father of the Prophet Muhammad (PBUH). Abu Talib also had two sons, Ali and Jaafar. Ali bin Abi Talib married Fatima, the Prophet Muhammad's daughter, and they had two children, al-Hassan and al-Hussein. And there are 37 generations between al-Hassan, the Prophet's grandson, and al-Hussein bin Ali, the Emir of Mecca (the King of Hijaz and Arabs). His Majesty King Abdullah II Ibn al-Hussein is descended from Al-Hassan, the Prophet's grandson. His Majesty is the forty-third generation to follow the Prophet Muhammad (PBUH) [9]. Before Islam, the Quraish arrived in Mecca in the second century AD. In the year 480, it seized control of Mecca under the command of Qusay bin Kilab. After him, it was ruled by his grandchildren from Bani Hashim. The Arab tribes admired them for their numerous good works in the sake of Arab unity at a time when battles raged between the fires of the East (Persians) and the fires of the West (Romans) [10]. The Hashemites' actions and responsibilities towards their nation prior to Islam were summarized in many ways, including organizing the Quraish tribe in particular and the people of Mecca in general, protecting the Ka'ba and its visitors, ensuring pilgrim comfort, and regulating trade and market matters. Mecca maintained substantial contacts with neighboring countries such as the Levant, Persia, and Abyssinia during the reign of the Hashemites. As Hashem enacted the winter trip to Yemen and the summer trip to the Levant, the tribes' hearts were filled with security and peace, and Hashem signed a pledge treaty with the Roman Caesar to protect Arab merchants traveling from the Arabian Peninsula to the Levant and vice versa, and the treaty was known as Elaf. The Holy Qur'an mentioned it in Surah No. 106 [11]. Many Hashemites believed in the Prophet Muhammad as a prophet of humanity, and they assisted him in spreading his call to the message of monotheism, while others did not. Even though he did not believe in him, his uncle Abu Talib stood by his side and shielded him from the polytheists. The Hashemites are divided into two groups: nobles and masters. The nobles are direct descendants of the Prophet Muhammad's eldest son, al-Hasan ibn Ali ibn Abi Talib, while the masters are the grandsons of al-Hussain ibn Ali ibn Abi Talib. Jordan's Hashemites are descended from the noble branch. Various noble families-controlled Mecca for nearly 230 years after Hijaz, but the Hashemite line, from which King Abdullah II originated, ruled from 1201 to 1925 [12]. As a result, the religious ruling authority in Jordan's Hashemite Kingdom enjoys legitimacy.
- *Historical legitimacy and its relationship to the Hashemites:* The Hashemites spearheaded a campaign advocating for conformity to God's law, the establishment of a shura among Muslims, and the administration of people's affairs. Many Hashemites were slain in the defense of the truth over time, which only strengthened their resolve and tenacity in their positions, eliciting admiration, appreciation, and respect in the hearts of Arabs in particular and the people in general. Sharif Hussein bin Ali, the creator of the Great Arab Revolt, was one of their most important roles that served Islam and the Arab country. Arabs unanimously agreed on Sharif Hussein bin Ali to be the Emir of Mecca, the head of religious affairs for Arabs, and the leader of the national movement after being subjected to oppression, persecution, confiscation, and torture at the hands of the Committee of Union and Progress, which came to power in the Ottoman Empire in 1908. Sharif Hussein consented to their plea for the sake of Arab independence, Arab unity, and the protection of Arab sanctuaries. On June 10, 1916, Sharif Hussein declared his revolution. The revolution went through three stages: the first was war in Hijaz, the second was battle in eastern Jordan, and the third was war in Syria [13]. The following are some of the most important factors that led to the selection of Sharif Hussein bin Ali as the leader of the national movement: his lineage to the Prophet Muhammad, his religious position as Sharif of Mecca, his extensive political experience, his belief in the Arab cause, freedom, and liberation, and finally, the strategic location of Hijaz, far from the Ottoman grip, where Sharif Hussein bin Ali lives. Among the causes of the Great Arab Revolt were the departure of the Committee of Union and Progress from Islamic teachings and its pursuit of Turkification policy, the spread of awareness among Arabs, political and intellectual in the Arab countries, and the deterioration of economic and political conditions in the Arab countries. The Great Arab Revolt was headed by a qualified Arab leadership led by Sharif Hussein bin Ali and his sons, the princes Ali, Abdullah, Faisal, and Zaid. Among the components of the Great Arab Revolt were distinct and definite goals, as well as British financial, political, and military assistance. Among the descriptions of the Great Arab Revolt are the following: a revolt was initiated against injustice in order to change Ottoman rule, which lasted for more than 400 years, most Arab countries engaged in it with around 100,000 fighters and covered huge sections of the Arab region [14]. During the four-year revolution, the movement was able to liberate Hijaz, eastern Jordan, Palestine, Syria, and Iraq. Although the revolution was broad in its opposition to Turkification, injustice, and persecution, it was not country, sectarian, or familial in nature, because Sharif Hussein spoke on behalf of all Arabs in his five correspondences with Britain in 1915 based on the Damascus Charter, which became known as the (Hussein - McMahon) correspondence [15]. For Sharif Hussein, the revolution was for all Arabs and only Arabs; as he put it, “the Arab revolution includes every Arab, whoever he is, as long as he is true to his country, loyal to his nation, and loyal to his people.” As a result, the historical reigning regime of Jordan's Hashemite Kingdom is legitimate [16].
- *Political legitimacy and its relationship to the Hashemites:* It is important to understand that the Great Arab Revolt resulted in the establishment of Arab kingdoms, including the Hashemite Kingdom of Jordan [17]. The revolution revived hopes for nationalism and Arab unity; and finally, the establishment of Arab kingdoms, including the Hashemite Kingdom of Jordan. By creating administrations in Hijaz, Iraq, Syria, and Transjordan, the Hashemites were able to build the Arab nation in political, economic, and social spheres. With the end of the Great Arab Revolt, Arabs overwhelmingly pledged fealty to Sharif Hussein bin Ali as Arab king, establishing the first monarchy in modern Arab history. Sharif Hussein bin Ali was distinguished by various traits, the most prominent of which were patriotism, adherence to the Qur'an and Sunnah, and a hatred of high positions. For him, ruling is a trust and obligation. On March 11, 1924, when Sharif Hussein bin Ali visited Amman, all Arabs vowed allegiance to him as Caliph of the Muslims and Commander of the Faithful [18]. Throughout history, the Hashemites have emphasized the importance of an Arab citizen's education and culture. The most important philosophical foundations of education in the Hashemites' era were, first, knowledge to purify morals and races, second, accustoming people to good behavior, third, understanding the basis of religion, and fourth, teaching the path of ancestors and Arabic literature. The Hashemites were also eager to improve the agricultural sector, believing that agriculture is the backbone of the economy, and so they attempted to retake and utilize some agricultural regions. They were also eager to develop educational and training facilities in order to provide Arab countries with educated and trained agricultural personnel. Throughout history, the Hashemites have used the concept of shura to govern [19]. Throughout history and throughout the many years of power, the Hashemites have taken care of Arabs, and this was reflected in the contemporary era by the governments founded following the success of the Arab revolution in 1920. Before, during, and after the Great Arab Revolt, four Hashemite kingdoms arose: the Hijazi Hashemite Arab Kingdom from 1916 to 1925, the Syrian Hashemite Arab Kingdom from 1918 to 1920, the Iraqi Hashemite Arab Kingdom from 1921 to 1958, and the Hashemite Kingdom of Jordan from 1921 to now. This study is on the Hashemite Kingdom of Jordan, which is the subject of the study. As a result, the reigning authority in Jordan's Hashemite Kingdom has political legitimacy [20].

**Second Factor**: the nature of Jordanian society:

Jordan has witnessed deep, extensive, and rapid social revolutions and formations since the founding of the state 10 decades ago, particularly in comparison to its Arab brethren and the human population in general. These changes are currently taking place. Jordanian civilization, like other societies across the world, is made up of families. In Jordanian society, two types of families exist: the traditional or old form known as the extended family or the large family or multi-generational family, and the modern or new form known as the nuclear or tiny family. The extended pattern is the most common family pattern and form in Jordanian society, followed by the nuclear pattern. However, it is currently the dominant nuclear pattern in Jordanian society's numerous urban, rural, and semi-urban groups [21]. Family ties in Jordanian society are strong and tight, marked by warmth, understanding, and mutual collaboration amongst individuals. One of the most important manifestations of the Jordanian family's strength and cohesion is the strength of relations between brothers and sisters, which stems from religious belief and close kinship; the strength of cooperation and respect between spouses in managing and taking care of family affairs, which is positively reflected in the upbringing and education of children to obey and respect their parents above all; as well as a strong relationship with relatives, particularly the husband's and wife's families, referred to as the “family of guidance.” Relationships here are typically formed through visits, invites, participation in wedding and funeral ceremonies, and the exchange of life counsel and assistance. All of these factors contributed to the formation of a cohesive and close society in terms of conventions, traditions, and conduct. This was represented in the discovery of a good citizen who belongs to and is loyal to his family, clan, tribe, region, country, and leaders. Jordanians have a particular personality that is defined by self-confidence, dignity, boldness, strength, courage, and generosity [22]. Jordanian society is a mosaic of social maps with different origins and origins. Despite the numerous and diverse population migrations to Jordan, Jordanian society has kept its customs and traditions, as well as its historical legacy passed down from parents and grandparents. Jordan experienced population migrations from many countries and regions prior to the establishment of the state; Jordanian society is a mingling of several components, including Circassians, Chechens, Kurds, Armenians, Turkmen, Druze, and Arabs from Palestine, Iraq, the Levant, Lebanon, the Arabian Peninsula, the North African Arab, and others; despite this societal and cultural diversity, Jordanian society is cohesive. They are all melted into the furnace of one Jordanian society under the auspices of real Hashemite Arab heritage [23]. Jordanian culture, like other societies, is made up of clans and tribes that are inextricably interwoven with one another by kinship or intermarriage, to the point where Jordanian society was virtually known as a one huge family society. The clan is regarded as one of the pillars of Jordan's security and stability. No matter how big the conflict among members of society is, it is settled tribally without turning to civil justice, and tribal judiciary is one of Jordanian society's inherited norms that everyone respects and values. Certainly, the Jordanian clan has contributed to Jordan's founding and construction, as well as to the system's preservation and stability. Jordanian tribes include all personnel of Jordan's armed forces, including the Arab army and security forces [24]. Since the founding of the Jordanian state, Jordanian clans have pledged to preserve the Jordanian state and its Hashemite leadership. The Jordanian slogan is “Jordanians of Belonging and Hashemites of Loyalty,” which demonstrates the Jordanian clans' love and dedication to the motherland and its leadership. Affiliation is the real and true affiliation with the homeland in thought and deed, pride in the homeland and dedication to its service and defense, participation in building and construction, preservation of achievements, voluntary work, knowledge of history and achievements, adherence to customs, traditions, and language, attention to national issues, and sacrifice for its sake. Jordanians of affiliation have an emotional value tied to the land. While loyalty is sincerity in adherence to the country's leadership and ruling system, and sticking by their side in the face of the country's hardships. Hashimite loyalty is an emotive attribute connected with leadership. The Jordanian clan is thus a crucial and political component, as well as the most fundamental pillar of Jordan's political stability [25]. Understanding, cooperation, and respect among Jordanians have resulted in unparalleled national unity. National unity is a popular consensus based on people's love for one another, and there is a strong family spirit. National unity is the most crucial pillar of the nation's foundations, development, and progress, as well as proof of the people's alignment with the leadership. Belonging and loyalty to the homeland, leadership, maintaining security, spreading love among the people of the country, rejecting violence, participating in the development of their homeland, maintaining its stability and achievements, cooperating between the citizen and the security man, and promoting the spirit of national dialogue among the segments of society are all components of national unity [26].

**Third Factor**: the rule of law and institutions:

The rule of law and institutions are the bedrock of future governments, particularly those seeking peace and safety at all levels for their citizens, stability for its entity and institutions, and progress for their development, renaissance, and structure. The rule of law and institutions are the final form of states that are capable of ensuring societal security through best practices in order to confront most of these global changes at their various political, economic, and social levels, as well as transcontinental transformations, particularly in light of the so-called creative chaos. There are two aspects to the term “state of law and institutions.” The first is that it is a legal state, meaning that it is governed and directed by its institutional, societal, individual behavioral, and organizational pathways. The correct and just laws and rulings that do not discriminate between any of the members of society, and are correct when faced with the violation of society's dissatisfaction and a demand Punishing those who deviate from it, and it is just when it does not discriminate in application, whether he is weak or strong, or between the poor and the rich. Only then can the state be described as a state of law and justice. It is an institutional state, or a state that is led, managed, mobilized, and ruled by collective and institutional controls, trends, and visions, rather than by individual or exclusivist whims, ideas, and opinions. As a result, state institutionalization is a continuous pattern of social conduct or a fixed technique of collective activity; it is a group of social relations established to employ and organize individuals' labor in order to attain common goals. What separates one society from another is the nature of its institutions, which are an intensification of its practices, norms, traditions, ideas, beliefs, laws, and degree of development, and the institution is, therefore, the cornerstone in the construction of civil society. They are legitimate organizations that have the ability to meet people's needs and defend their rights over time. The process of enacting laws in a state is regarded as the rules that structure society rather than those that regulate and govern it. Laws, on the other hand, govern the interaction between society and the general system of the state, as well as the sovereign state itself within a group of states or throughout the global system. As a result, the state of institutions, defining responsibilities, and the rule of law is the state of civil society, discipline, and human organization in order to form human humanity and group cohesion and unity, rather than a forest ruled by an individual or even authoritarian institutions and individuals who are unique in opinion, ideas, and responsibilities, and who are driven only by chaos, force, whims, and personal or factional interests. This has nothing to do with the existence of a ruling authority, a higher authority, or a government that oversees these institutions, laws, and individuals. Rather, it is a rational, logical, organizational, and administrative necessity that is unavoidable in any institutional state and modern civil society that seeks to achieve the best ways of life for its members, provided that the role of the government or authority and its main roles in this institutional building are limited to providing the means to amend laws and settle disputes about the meaning of laws, as well as to carry out its commitment. In short, the concept of state of law and institutions emphasizes the transition of the state from the authority of the individual, group, or center to the authority of the institution, and from traditional society to civil society, and from a state governed by the laws of power, class, and individual authority to a state in which power is distributed and shared, not on individuals or groups or groups, but on legislative, executive, and judicial authorities. If society manages to move itself to this advanced stage of civility, modernity, and democracy in which the rule of law and institutions triumph over the authority of groups and individuals through free will, it will be stable, secure, and assured of its present and future. The future state will be built on the rule of law and institutions. Jordan is a state of law with well-established institutions that ensure everyone's rights, and Jordanians' commitment to using the law and the impartial Jordanian judiciary comprises the greatest level of national consciousness in controlling all aspects of their life. And when we discuss the rule of law and institutions, we are discussing the rule of law. And when we talk about the rule of law and its respect, we are talking about the behavior of the citizen or any resident in Jordan, as well as the behavior of that employee, policeman, minister, or any other person in a position of responsibility, when he proceeds to apply the law to himself, similar to developed and civilized countries with values, law, and everything, because the law is one. It is critical to recognize an unavoidable fact: the rule of law and respect for it do not stem solely from the actions of official authority. The desire of any free and honest official or citizen to belong to the nation is the source of Jordan's rule of law. Because the rule of law achieves the ideas of transparency, justice, and equality of opportunity for any Jordanian citizen who has the citizenship rights granted by the Jordanian constitution. This necessitates officials being up to the level of responsibility and His Majesty's thought that the country's capabilities are a trust and responsibility, and that they must understand that they are there to serve the country and the citizen, and that the law takes its course in holding every corrupt official accountable and trading in the interests of the country and citizens, and manipulating laws and Jordan's capabilities according to his own mood, whether h Jordan is who he is now. As a result, the rule of law and institutions have contributed to Jordan's political stability over the last century. Political stability can be defined as a political system's ability to manage internal conflicts within the framework of the law, to successfully deal with crises, and to adapt and respond to political developments. In this context, Jordan has withstood as a political entity, despite its presence in an unstable region, but it has not witnessed complete political stability, with what this concept includes of indicators and manifestations, since the establishment of the emirate in 1921, but rather relative stability due to the region's political, security, social, and economic events and developments [27].

### 3.2 The second axis: the stages of political development in Jordan

Before delving into Jordan's political stages, it is vital to first comprehend Jordan's founding stage. Following the battle of *Maysaloun* in 1920, Sharif Hussein bin Ali pleaded with Syrian Arabs to rescue and help them. On April 11, 1921, Prince Abdullah bin al-Hussein established the Emirate of Transjordan and established the Council of Advisors, with Rashid Ṭaliʽa designated as Jordan's first prime minister [28]. At the time, the emirate had three districts: *As-Salt, Ajloun, and Karak*, but it today has 51 districts spread among 12 governorates. On May 25, 1923, Britain acknowledged Transjordan's independence, and in 1925, Prince Abdullah issued a royal will to annex the territories of *Ma'an and Aqaba* to the Emirate of Transjordan after his brother, King Ali bin Al-Hussein (King of Hejaz), ceded them in the Jeddah Treaty on June 5, 1925 [29].

The three stages that have played a critical role in Jordan's political evolution are the foundation of the emirate (1921–1928), the Jordanian-British Treaty of 1928, and the Independence stage in 1946. The three stages are discussed in the following order:

**First Stage:** *the establishment of the emirate 1921–1928:*

This stage was known as the pillars of the new state stage, and it included centralizing the new administration, developing an army to strengthen the government, and training the population to accept the new emirate. This occurred without significant clashes between the state and the people, so the government became centralized under one leadership after there were multiple leaders on Jordanian land, and the army was built from all Jordanian people components, and the people were prepared to accept the new emirate with a flexible discourse [30].

**Second Stage** *: the Jordanian-British Treaty of 1928:*

The formation of a basic legislation for the state, Britain transferring executive and legislative authority to Prince Abdullah, and the supply of British aid to eastern Jordan were the most important developments at this time and in the treaty. In light of this, the Basic Law (Constitution) was promulgated in 1928, establishing a Legislative Council. Jordan's first election law was passed, and elections were held, resulting in a Legislative Council of 16 members in April 1929, including nine Muslims, three Christians, two Circassians, and two members appointed by Prince Abdullah, one from the Bedouins of the north and one from the Bedouins of the south. Today, the House of Representatives has 130 members from various walks of life in Jordan.

**Third Stage:** *The Independence in 1946:*

Jordan, as previously stated, was created in 1921 as the Emirate of East Jordan, and then became known as the Emirate of the Arab East in 1925, until settling on its current name in 1946 following independence as the Hashemite Kingdom of Jordan. On May 25, 1946, the Jordanian kingdom was declared totally independent by the Fifth Legislative Council, who vowed loyalty to the heir of the Arab Renaissance, Prince Abdullah bin al-Hussein, as a constitutional monarch. The kingdom's second constitution was promulgated on February 1, 1947, and parliamentary elections were held on October 20, 1947. The elections produced the first parliament in the fledgling kingdom's history [31]. Jordan (the Hashemite Kingdom of Jordan) has seen four kingdoms since its inception in 1921, one of which was during the emirate era before independence and three during the kingdom era following independence. This study focuses on the four kingdoms and the most significant events that each of these four kingdoms observed, as follows.

- *The First Kingdom: 1921–1951:* The first kingdom is distinguished by the fact that it is a kingdom of two covenants, the princely and royal covenants led by King Abdullah I bin al-Hussein. The first kingdom witnessed many stations and many achievements, which can be summarized and combined as follows: the first kingdom witnessed two independence, the first during the emirate's era on May 25, 1923, and the second during the kingdom's era on May 25, 1946; the name of the Arab East for the Emirate of East Jordan in 1925; the issuance of the Basic Law in 1928; the first parliamentary elections in 1929, and referring to the Arab Army (the Jordanian army's official name until today) as the All-Arab Army; The first significant conflict between Arabs and Israelis occurred in 1948, which is known as the Nakba year. Participation in the foundation of the League of Arab States in 1945; dedication to the objectives and ideas of the Great Arab Revolt, which demand and accomplish Arab unity; Furthermore, on July 20, 1951, King Abdullah I was assassinated at the holy al-Aqsa Mosque in Jerusalem. The late King Abdullah I Ibn al-Hussein, founder of the Hashemite Kingdom of Jordan, can be described as a one-of-a-kind figure in the history of the modern Arab world. His persona was a cross between traditional and modern. Throughout his public life, he was a modern, forward-thinking man. This was manifested in him because he was one of the first Arab presidents to establish a constitutional monarchy following Jordan's founding, as evidenced by his practical experience and interaction with his people [32]. During the Arab revolution, the founding king commanded the Arab armies under the Hashemite banner, inspired by the beliefs of his father, Sharif Hussein bin Ali, and his brothers Ali, Faisal, and Zaid. The Great Arab Revolt liberated Damascus, modern Jordan, and the majority of the Arabian Peninsula at the end of World War I. In 1921, King Abdullah I formed the Emirate of Transjordan, establishing the first central government structure in a predominantly tribal and Bedouin nation. Over the next three decades, he concentrated on state-building and the institutional framework of contemporary Jordan. The King sought sovereignty and independence with determination and a forward-thinking vision, building democratic legitimacy by drafting Jordan's first constitution in 1928 and holding elections for its first parliament in 1929. During these three decades, the King concluded a series of treaties with England and Transjordan, the last of which was the Anglo-East Jordanian Treaty on March 22, 1946, which ended the British Mandate and gave Transjordan full independence, and the country became known as the “Hashemite Kingdom of Jordan” on May 25, 1946 [33]. Jordan began to play an advanced Arab and international role and participate in conferences after achieving complete independence, the first of which was the *Inshas* summit on May 28, 1946, days after the state's independence, and Jordan then assumed an advanced position in the service of Arab causes, the most prominent of which is the Palestinian cause. During the 1948 Arab-Israeli War, the Jordanian Arab Army played an important role in defending Jerusalem and other portions of Palestine. The Jordanian army displayed bravery and heroism, and was well-known for its professionalism, steadfastness, and fortitude in the face of a force superior in numbers and equipment. The Jordanian Arab army defeated Jewish forces in *Bab al-Wad, al-Latrun*, and Jerusalem, and East Jerusalem was preserved despite following massive Israeli offensive that failed to wrest it from the Jordanian Arab army. The conflict concluded in mid-July 1948, and at the Rhodes Conference in 1949, a variety of armistice agreements were made between Arabs and Israel. According to them, the Transjordan region's borders with occupied Palestine were demarcated. On July 20, 1951, the late King Abdullah I went to Jerusalem to perform Friday prayers with his young grandson, Hussein bin Talal; however, fate had other plans for him, and the king died as a martyr at the threshold of al-Aqsa Mosque and near the tomb of his father, Sharif Hussein bin Ali, who sacrificed for all Arabs. The young prince, King Hussein Ibn Talal, was by his grandfather's side at the time, observing the scene and a bullet falling from Medal's breast. His Majesty was there at critical junctures throughout the events. The assassination of his grandfather, King Abdullah I, had a major impact on his life in terms of his knowledge of the seriousness and inevitability of death, as well as his subsequent feeling of duty and responsibility. In his book, “Problems of Kings,” King Hussein writes that three days before that fateful day in Jerusalem, his grandpa turned to him and said, “I hope you realize, my son, that you will take charge one day.” Do your utmost to ensure that my efforts are not in vain. I hope you will continue to serve our people” [34].
- *The Second Kingdom: 1951–1952:* The second kingdom is distinguished by its brief duration, only one year, yet it has accomplished much under the leadership of King Talal bin Abdullah. The Second Kingdom witnessed many stations and achievements, which can be summarized and combined as follows: the issuance of the Jordanian Constitution in 1952, which is considered one of the most recent constitutions in the world and is still in force today; and the establishment of the Audit Bureau in 1952 to monitor state revenues and expenditures; compulsory and free primary education; and adherence to the goals and principles of the Arab League. King Talal bin Abdullah took control shortly after his father, King Abdullah I, was assassinated on July 20, 1951. However, due to health issues, he was unable to continue to govern, and he was deposed less than a year later, on August 11, 1952, and King Talal accomplished much during his term to build connections between Jordan and its Arab, regional, and international surroundings. King Talal was also primarily responsible for the development of a new modern constitution that held the government collectively and ministers individually accountable to Parliament, which was ratified on January 1, 1952.
- *The Third Kingdom: 1952–1999:* The third kingdom is distinguished by its long 47-year history, which has seen significant successes under the leadership of King Hussein bin Talal. The Third Kingdom saw several stations and accomplishments, which can be summed and merged as follows: The Arab Army was Arabized in the United Kingdom in 1956, following the removal of British General John Bagot Glubb from command and the appointment of Jordanian Major General *Radi Annab* as Chief of the Joint Chiefs of Staff; and the second major Arab-Israeli war in 1967; the occupation of the West Bank and East Jerusalem from Jordan, the Golan Heights from Syria, the Gaza Strip and the Sinai Peninsula from Egypt; and the Battle of Karama in 1968. Israel launched a violent battle against Jordan on March 21, 1968, as part of its expansionist intentions, but the Jordanian army met them with heroism, manliness, and redemption. The Army was able to overcome Israel and defy Israel's long-held belief that their army is unbreakable. The United Kingdom project was presented in 1972 between Jordan and the West Bank/as a new nucleus towards Arab unity/and the most important items of the project were: the establishment of a united Arab kingdom consisting of the Jordanian state with Amman as its capital, the West Bank, and any future liberated Palestinian territories with Jerusalem as its capital. Assuming that Amman is the capital of the United Arab Kingdom, and that the head of state is the King of Jordan, who controls the country through a central executive body, and that the National Assembly is elected by secret ballot with equal representation from both parties. The United Arab Kingdom shall have armed forces whose supreme commander is the king, and each party shall have a general governor and a council of ministers from its individuals, but the project failed; and the Ramadan/October/Yom Kippur (Yom Kippur) war in 1973, one of the results of which was the Arabs' meeting on one goal, which is to fight Israel, the return of the Palestinian cause to international forums, and the Arabs' use of the oil weapon against the West. The most crucial aspect of the stage was the West's consideration of peace between the Arabs and Israel in order to weaken them. After the signing of the Camp David Treaty in 1978, Egypt was removed from the arena of the Arab-Israeli conflict; the third kingdom focused on education, as it witnessed the founding of the first university in 1962, the University of Jordan, the mother of Jordanian universities; the administrative and legal disengagement with the West Bank in 1988 at the request of the Palestine Liberation Organization after it became the only legitimate represent of the Palestinian people. However, the decision to disengage did not include Jordan's continued care for Islamic holy sites in Jerusalem; the resumption of parliamentary life in Jordan in 1989, and the holding of parliamentary elections after parliamentary work in Jordan had been suspended since 1967 due to the occupation of the West Bank; and the signing of the Jordanian-Israeli treaty, the Wadi Araba Agreement, on October 26, 1994. According to this agreement, Israel's borders were set, effectively ending the Israeli dream in Israeli borders stretching from the Euphrates to the Nile. King Hussein bin Talal will be remembered and remembered as a king who guided his country through crises and instability until Jordan became a haven of peace, stability, and moderation in the Middle East. Jordanians regard his memory with respect and appreciation as a source of inspiration for Jordan's climate of openness, tolerance, and empathy. King Hussein has left a flourishing legacy that will steer Jordan for many years to come. His Majesty enjoyed the longest reign of any world leader when he died on February 7, 1999. King Hussein bin Talal was proclaimed king of the Hashemite Kingdom of Jordan on August 11, 1952, but because His Majesty had not yet reached the age of eighteen, a Regency Council was formed to take over the administration of the country until His Majesty's constitutional entitlement to His Majesty's assumption of his constitutional powers on May 2, 1953, when the ceremonies of handing over His Majesty were held. King Hussein then immediately focused on constructing an economic and industrial infrastructure to supplement and enhance the advances he desired to accomplish in terms of his people's quality of life. Jordan's key industries, such as phosphates, potash, and cement, grew during the 1960s. A road network was built to connect all sections of the Kingdom. The stats speak about King Hussein's human achievements. In 1950, only 10 % of Jordanians had access to water, sewage, and electricity; today, this figure has risen to 99 %. The number of literate persons increased to 85.5 % in 1996, up from 33 % in 1960. According to UNICEF figures, Jordan had the world's fastest yearly rate of decrease in under-year mortality from their age of 70 deaths per 1000 live births in 1981 to 37 deaths per 1000 live births in 1991, a drop of 47 %. Jordan's most valuable possession, according to King Hussein, has always been its people. Throughout his reign, he urged all citizens, even the less fortunate, the crippled, and orphans, to strive for greater accomplishments in order to better serve themselves and their country. Throughout his forty-seven years of power, King Hussein has also struggled to bring peace to the Middle East. He played a key part in the formulation of Security Council Resolution 242, which was issued following the Arab-Israeli war in 1967. In exchange for peace, this resolution demands that Israel depart from all Arab countries it conquered during the 1967 war. This decision served as the foundation for all following peace talks. In 1991, King Hussein was instrumental in calling the Madrid Peace Conference and in establishing an “umbrella” under which Palestinians could negotiate their future as part of a Jordanian-Palestinian delegation. Jordan and Israel signed a peace deal in 1994, which is a significant step toward attaining a just, comprehensive, and long-term peace in the Middle East. King Hussein also attempted to bring Arab countries together. During the Gulf War of 1990–1991, he worked tirelessly to guarantee a peaceful Iraqi departure and the restoration of Kuwait's sovereignty. The Jordanian government produced a white paper outlining the Jordanian leadership's true and sensible perspective on the Gulf crisis. His pursuit of genuine Arab reconciliation served as a catalyst for mediating the Yemeni civil war. Furthermore, in the majority of his speeches and from the majority of pulpits, His Majesty called for international humanitarian help to alleviate the everyday suffering of the Iraqi people. Jordan has been a regional role model due to King Hussein's devotion to democracy, civil liberties, and human rights. At a time when recent reforms have opened the path for Jordan to restart its irrevocable democratic option [35], the kingdom enjoys international acclaim for its human rights record in the Middle East. In 1990, King Hussein appointed a royal committee representing all spectrums of Jordanian political thought to draft the National Charter, which is now regarded as guidelines for the country's process of democratic institutionalization and political pluralism, alongside the Jordanian constitution. Jordan held parliamentary elections in 1989, 1993, and 1997 that were widely regarded as among the freest and fair in Middle Eastern history [36].
- *Fourth Kingdom: 1999 - to the present:* The march has continued in the fourth kingdom since 1999, led by His Majesty King Abdullah II Ibn Al Hussein. His Majesty King Abdullah II took office on February 7, 1999, following the death of his father, the late King Hussein bin Talal. This was known as the Day of Loyalty and Allegiance; allegiance to the late great King Hussein and allegiance to King Abdullah II. Many stations and achievements occurred during the Fourth Kingdom, which can be summed and consolidated as follows: Jordanian youth, who make up 70 % of Jordanian society, were given more space in the Fourth Kingdom, pushing them to join in constructing and giving and to be active partners in building modern Jordan. His Majesty referred to them as the Knights of Change; and launching an initiative in accordance with the measure of the people's determination, given the latent energies possessed by Jordanian society in order to invest it in the progress of the Jordanian construction wheel; and launching the Jordan First Initiative in the year 2002, because Jordan is part of a whole, it is an integral part of its Arab system. On the foundation of the nation-state rather than the country-state; and the 2006 launch of the “We are all Jordan” effort to eliminate the phenomena of societal violence in Jordan, which may tamper with the national fabric and undermine national unity, which is the state's cornerstone of progress; and the 2004 introduction of the Amman Message project to enlighten the world about Islam's tolerance and acceptance of the other; The establishment of the Ministry of Political Development to activate Jordan's political parties and enable them to take their natural place in democratic political work pushed the wheel of political parties forward, and encouraging internal and external investment, in order to make Jordan an appealing investment destination, to create job opportunities for Jordanians, and to address the problem of unemployment, which causes poverty; and allowing the popular movement to freely express its flag in its demand for reform, the Kingdom's response was to amend the Jordanian constitution, establish a constitutional court, and an independent body to supervise parliamentary elections; His Majesty King Abdullah II also launched the World Interfaith Harmony Week initiative in 2010, which was proposed by King Abdullah II at the United Nations in 2010 to promote cultural harmony. The United Nations General Assembly endorsed World Interfaith Harmony Week in resolution No. 65/5 on October 20, 2010 and declared that the first week of February of each year will be observed as a global week promoting interfaith harmony. The General Assembly highlighted in its decision that mutual understanding and interreligious dialogue are two fundamental components of a global culture of interreligious peace and harmony, making World Week a way of promoting concord among all people regardless of religion. Recognizing the urgent need for interreligious dialogue and promoting mutual understanding, harmony, and cooperation among people, the General Assembly encourages all nations to support this week's efforts to spread the message of harmony through churches, mosques, temples, and other places of worship, on a voluntary basis and in accordance with their own religious convictions and traditions [37].

### 3.3 The third axis: the Hashemite Kingdom of Jordan's place on the political map of the world, regionally and internationally, in the first centenary of its modern era

Jordan's progress, modernity, and expansion throughout the past century, despite all of the problems it has faced, whether linked to its geographical character and people resources, or political or economic, has made this country the focus of everyone's attention as a role model [38]. The process of constructing, developing, and modernizing the state illustrates Jordan's strength in achieving a blend of modernity and the positive customs and traditions that characterize this country, its leadership, and its people, of tolerance, kindness, moderation, and commitment. Jordan surmounted all of its obstacles, political, economic, and security, by relying on factors that constitute an integrated cycle that shaped the contemporary state. Jordan's only support came from the Hashemite leadership's recognition of the importance of Jordan continuing to meet obstacles via affirmation and commitment to achieving democratic legitimacy. Jordan's stages, from its establishment in 1921 to today 2021, represent a state that is advancing in an advanced, forward-thinking manner, overcoming all the obstacles in its road with the zeal of Jordanians and Hashemite leadership. The observer of these stages notes significant points in Jordan's history that took it from the construction period to the stage of growth, and then to the stage of development and modernity. Jordan's succession to the throne of His Majesty King Abdullah II was celebrated on June 9, 1999, when the flag was given to him following a hectic era sponsored by the late King Hussein while developing state institutions. On February 7, 1999, His Majesty King Abdullah II took the oath of office, promising loyalty to the motherland and the constitution in order to maintain it. Every Jordanian signed an oath of loyalty to the country and the monarch, the preservation of the constitution, and advancement toward success. His Majesty begins a new stage, which is a comprehensive revolution in the method of labor and management, as well as how to integrate all segments of society in progress, on that day. His Majesty's non-stop initiatives called on everyone to be creative and self-improvement in order to improve the quality of life, with the realization that the march of progress in the world does not stop and does not wait for anyone, and that we must keep pace with the times in order to enter the stage of the modern global state with open thought, moderation, and success was consolidating the will for change, so that the will for change became a Jordanian national ascription [39]. His Majesty King Abdullah II believes that the new stage necessitates reaching out to the world and opening up to it, introducing Jordan and working under the sun while maintaining the constants and values that have been firmly established in a Jordanian Arab Islamic society whose identity is clear and deep Arabism. His Majesty's attention is dedicated first on the Jordanian citizen in Jordan's homeland. Training, education, and rehabilitation were the emphasis of all development programs, and His Majesty advocates for investing in human resources and rehabilitating Jordanians because it is the foundation for the success of investment in its entirety. His Majesty focuses on the Jordanian human being by emphasizing the youth, who account for more than 70 % of Jordan's population. Almost every speech or talk given by His Majesty addresses young people and emphasizes the importance of their participation in developing and improving society. His Majesty is eager to attend their conferences and forums and engage in dialogue with them. It arrives at their schools and universities and permits them to attend worldwide meetings alongside His Majesty. Jordanian youth have been a notable presence in the Dead Sea Economic Forum on multiple occasions. “It is necessary to focus on the youth component, care for them, activate their role, and participate in the development process, bring about change, and build the future, within a clear comprehensive vision, and work in the spirit of a single team,” His Majesty said. Belonging … Belief in his message … His vision and capacity to accomplish goals” [40]. Jordan is proud of its youth institutions, which are expanding their coverage base for all young people on a daily basis to match the implementation of youth plans and policies aimed at integrating them into long-term development. Jordan and its leadership consider the future and the younger generation, and how to realize their hopes, as well as how to overcome the difficulties in front of them and provide them with quality education; because they believe that this matter will contribute to reducing poverty and unemployment, as well as supporting and strengthening initiatives to develop Jordan and raise the Jordanian people's standard of living. Because youth constitute the backbone of the educational process, His Majesty's commands are continuously geared toward educational growth. His Majesty initiated the Education Development Initiative to improve the educational system's capacities and position Jordan as a global source of expertise in the electronic system and the knowledge economy. As a result, Jordan has institutes that teach trainers in the field of technology education. According to UNESCO figures, Jordan ranks eighteenth out of ninety-four countries in terms of educational attainment. Jordan is interested in the generalization of e-learning because it has completed the computerization of education project, which included introducing this modernization and development to thousands of schools, as well as completing the construction of a fiber-optic network with the goal of adopting information technology to include schools, universities, and community colleges, with private sector participation. A Jordanian educational institution does not lack information technology or specialized courses, and this knowledge has become a basic entrance point for all sciences [41]. In the sphere of education, the royal initiatives have sparked interest in delivering education and assisting students through the King Abdullah II Fund for Excellence, which has branches in various Jordanian universities. The fund's concept is based on the premise that education is a right for all, and that material circumstances should not hinder certain students from benefiting from educational opportunities and from displaying their potential. And His Majesty's directions to secure suitable housing in many rural locations, which have now become model villages, as well as finding projects in the same places to give job opportunities for locals and defend the Jordanian middle class [42]. Jordan is seeing rising growth in the fields of economy, industry, trade, and health, as evidenced by an increase in capital coming to Jordan to invest due to its good facilities, stringent legislation, security, and stability. In the integrated cities that will transform the face of Aqaba, and investments in them will surpass one billion Jordanian dinars, in addition to infrastructure projects in Jordanian governorates, and the establishment of development zones in numerous governorates around the Kingdom. The intra-Arab trade movement is active, particularly since the signing of the intra-Arab trade pact in which Jordan participates, and statistical data indicate real growth in the Jordanian national economy. Jordan pays special attention to debt, which it deals with in an open and credible manner, as evidenced by the volume of foreign trade. Since taking office, His Majesty King Abdullah II has prioritized human resource development, raising the level of basic government services, accelerating the implementation of major national projects in water, energy, and infrastructure, involving national capital in them, implementing anti-poverty programs, and creating opportunities. He aimed to minimize unemployment through governorate development programs, to encourage small projects, and to promote governorate development. His Majesty delegated broad powers to the governors to oversee the implementation of development plans. His Majesty King Abdullah II is having a successful world tour, explaining the reality of the Jordanian economy and Jordan's aspirations, which include a comprehensive plan for an economy that will attract foreign investment and the job opportunities that these investments provide for Jordanians. Among the aims of development plans are the development of governorates and the sharing of development gains. His Majesty's initiative was to divide the Kingdom into three development regions, with elected development councils and elected local government bodies (municipalities) in addition to one political parliament for the state, in order to achieve a state of interaction and focus on implementing development and progress plans, with an emphasis on decentralized administration and regions, with the goal of expanding the base of popular participation in decision-making and priorities. His Majesty said: “Our vision is to achieve a modern Jordan that meets Jordanians' aspirations for progress and prosperity.” Efforts in the public and private sectors, the National Assembly, and civil society institutions in the media and press must be harmonized and combined in order to develop a complete agenda that includes national goals that symbolize everyone's vision.” Defining strategic programs and national strategies, the fulfillment of which will be required of subsequent governments [43]. His Majesty emphasizes the necessity for reform, as a brief examination of the recent and distant past reveals that the state's trajectory requires interventions to free it of confusion, unpredictability, regionalism, and narrow perspectives. The National Agenda addresses the subject of how Jordanians might work together. It is an agenda that accepts only a real national vision, does not tolerate interpretations of who is Jordanian, does not accept a concept of a homeland other than its fully Jordanian, and does not accept any meaning for identity other than the one Jordanian national identity. The National Agenda was created to meet the difficulties that the Kingdom will confront in the many developmental and political areas in the future. It is a unified, consensus-based national vision that looks forward to the future and prepares the country to face changes and avoid shocks. The royal vision for the media comes to confirm its independence and to encourage a national media with improved press freedoms. His Majesty's directions in support of media institutions were to be administered with a new vision and philosophy aimed at developing a new media model, with an emphasis on press freedom and ensuring that sufficient space for speech is provided by legal legislation to attain a responsible media. The nation and asserts its identity, as well as understands the meaning of Jordan first, the substance of the national agenda, and aspirations of comprehensive reform that originate from within [44]. The Amman Message, which explains Islam's approach based on respect for human values, condemns violence and extremism, calls for dialogue, and highlights Islam's true image based on the foundations of goodness, justice, tolerance, and moderation, was a message that found understanding and acceptance from all global circles. The speech embodied Islam's major ideals of charity and honor for humanity, human friendship, respect for pacts and covenants, and asking the nation's intellectuals to do what exposes the truth of Islam and its lofty values. Jordan was able to establish a presence at the regional and international levels under Hashemite rule, particularly in its defense of its national issues, particularly the Palestinian cause, which is its central issue, in addition to Jordan standing by all Arab brothers and supporting their causes. The Hashemite leadership's policy of transparency, openness, and moderation in the proposal was one of the main factors that made Jordan the focus of international and global attention in proposing and presenting visions and ideas to meet the challenges and overcome the obstacles confronting the region. Here, we recollect His Majesty King Abdullah II's historic address before the United States Congress, which exemplified Jordan's philosophy of honesty, clarity, and moderation in championing the causes of his country and mankind as a whole. Jordan's presence in providing humanitarian help to people all over the world symbolizes the country's civilized and humanitarian role. Jordan's march from its formation and independence to the present displays Jordanian prosperity, which Jordanians have made dear and valuable in order to rule it under a distinct and wise Hashemite leadership. Jordanians are proud today of the Hashemite leadership for laying the foundations of the modern Jordanian state and consolidating the features of freedom, democracy, and institutionalism, despite changes and regional and international challenges. As Jordan demonstrated a model for serious work toward solving the issues and challenges confronting Jordan and the Arab nation during His Majesty King Abdullah II's reign, in addition to His Majesty's great work in developing political, cultural, economic, and security aspects, as well as working to build the armed forces on modern foundations and with a high level of professionalism [45]. The Hashemites had a wide, large, and numerous roles in the construction of the Jordanian state, and they exercised their obligation towards the holy places in Jerusalem, based on the historical Hashemite guardianship over Islamic and Christian holy sites. Because of their relationship to the Arab Hashemite Prophet Muhammad, the Hashemites were historically committed, generation after generation, to a lawful contract with Islamic sanctities, therefore they safeguarded and protected their status and cared for them. Today, Jordan stands firm in the historical legacy carried by the nation's hope and pride, His Majesty King Abdullah II, who worked to develop the concept of the state and push it to move towards the technological and scientific developments of the era in line with His Majesty's wise vision to achieve Jordan's ambition and more comprehensive openness between the leadership and the people. Through the royal discussion papers, which plainly demonstrate His Majesty's total commitment to achieve the rule of law, production, and social solidarity.

Although Jordan maintained its stability during the first centenary, at the beginning of the second centenary, Jordan faces internal and external challenges and threats that constitute a major existential threat to its security and stability, as decision makers cannot ignore them. Regardless of the regional and international competition over the Middle East, successful political experiences must be adapted in order to mobilize citizens behind their leadership and government to enhance security and stability at all levels to face challenges and threats. In addition, a network of complex, multidimensional relations of regional and international alliances would ensure the achievement of Jordan's supreme national interests. Perhaps expanding and diversifying the network of regional and international relations is necessary if we take into account the economic crises that Jordan has been going through for several years, in addition to the marginalization of Jordan's central geographical role in the region. However, the imbalances in the economic situation negatively affect development. This is what prompts Jordan to reconsider its position and to assert its regional and international importance. Therefore, these challenges can be divided into three categories: short-term challenges, medium-term challenges, and long-term challenges. One of the near-term challenges, for example, is the problem of unemployment, as more than 70% of the Jordanian population are young people under the age of 40 who have the ability and competence to work, but they cannot find opportunities that are compatible with their studies and experiences. The problem of poverty is also one of the short-term challenges facing Jordan, which has become chronic due to imbalances between financial and economic resources on the one hand and the increase in population as a result of waves of refugees from Syria, Iraq, Palestine, Yemen and Libya on the other hand. In Jordan, there are more than 55 Arab and foreign nationalities. Also, the population growth rate is 3%, which is one of the highest rates on a global level. This is a major challenge that should be addressed urgently with wisdom and a high sense of responsibility [46]. As for the medium-term challenges, for example, the water problem, Jordan has not succeeded over the past decades in finding long-term solutions to the water scarcity crisis, at a time when Jordan is classified, according to the global water index, as the second poorest country in the world with water. And because water is life, and the agricultural, industrial, tourism and other sectors depend on it, these sectors will inevitably compete with people over drinking water. As part of efforts to mitigate the crisis, an agreement was signed between Jordan and Israel, which reached an agreement whereby the latter would sell 50 million cubic meters of water annually to Amman, and the agreement was signed in October 2021. This in itself constitutes another challenge for Jordan that may affect its security, political and sovereign situation in the future [47]. The water insufficiency narrative blames factors external to the government or Jordan's circle of influence and responsibilities: immigration and refugees' influx; external countries and regional political situation; climate change; and nature and environmental conditions [48]. As for the long-term challenges, for example, the problem of the Israeli-Palestinian conflict. Jordan considers the Palestinian cause its first central issue, and views it as a priority in its foreign policy, and considers it a central and fundamental issue for the security of the region, the solution of which is the key to peace and stability in the world. Jordan believes that the establishment of an independent Palestinian state represents a supreme Jordanian national interest, and that a comprehensive and just peace will only be achieved by meeting the legitimate aspirations of the Palestinian people and establishing their independent state according to the principle of the two-state solution. His Majesty King Abdullah II always stresses that the region will not enjoy security and stability unless the Palestinian issue is resolved, in a way that achieves the establishment of an independent Palestinian state with East Jerusalem as its capital, within the framework of a just and lasting settlement. Without this solution and without ending the occupation, there will be neither peace nor security in the region. Jordan will continue to work to find a just and lasting solution to the conflict in which the two-state solution will be embodied and the establishment of an independent Palestinian state with Jerusalem as its capital on the lines of June 4, 1967, in accordance with the resolutions of international legitimacy and the Arab Peace Initiative, and in a manner that guarantees a solution to the five final status issues in which Jordan has direct interests (Jerusalem, refugees, borders, water and security) [49].

## 4 Methodology

### 4.1 Study's approaches

The study used the historical, descriptive analytical, systemic analytical and decision-making methods for the purposes of answering the study's questions and testing its hypothesis.

### 4.2 Study's area

This study is part of the Jordan Celebrations Project in its first centenary, which covers a period of time that extends between the year 1921–2021. The year 1921 marks the beginning of the establishment of the Jordanian state. Where Jordan witnessed, over the course of 100 years, many local, regional and international challenges that had an impact on the path of the state, but it was able to face these challenges with security and peace due to the factors that the study talked about in the axes that were mentioned above in the literature review. As these factors have contributed to the stability of the political system in the Jordanian state, despite the stormy stages that afflicted the region in a continuous manner that overthrew many political regimes.

### 4.3 Study's data collection

Experts with historical, political and social knowledge and experience were consulted in proposing this project. Where the idea of the project was discussed with various working and retired segments of senior civil, military and security officials and academics, as the idea of the project was welcomed and encouraged by all, which encouraged the researcher to proceed with work on completing the project coinciding with the celebrations of the Jordanian state in its first centenary in October 2021. The researcher was able to collect data related to the project through: interviews with people with experience and knowledge, related studies and research, and access to official sites in the Jordanian state, including the Royal Court, the Prime Ministry, the Ministry of Foreign Affairs, the National Library and the National Documentation Center. The project was reviewed and audited with some of the experienced and knowledgeable companions, and after that the project was submitted in its final form to the journal for publication in a timely manner.

### 4.4 Study's analytical framework

The analytical framework for this study is derived from the Structural, Role and Systems theories as well. The structural theory of political stability has many meanings, all of which relate to the existence of a state of regularity in the course of affairs in any country, according to prior planning by the political system, so that things proceed in a manner consistent with what is planned by the political system, and therefore its returns are calculated and often What is in its favor, and this is what the Jordanian state has pursued since its establishment by following the policy of planning and organizing in search of building a state of law and institutions, which has been achieved over the past ten decades. The role theory is one of the modern theories in sociology, and it believes that the behavior of the individual and his social relationship depends on the role he plays within society, according to the rule of rights and duties, this inevitably contributes to strengthening trust between the individual and the state. The theory of systems that is based on inputs, processes and outputs, and this is what the Jordanian state has adopted since its establishment until now, as the Jordanian state was keen to keep pace with all that is new in terms of development and modernization, the latest of which was the system of political, economic and administrative modernization ordered by His Majesty King Abdullah II at the beginning of the second centenary, which was adopted and became a clear approach in early 2023.

This study conducted a thematic analysis of the information gathered from the experts as well as from the aforementioned studies and sites related to the project in the Study's data collection, and then used the aforementioned three theories in study's analytical framework, in processing the gathered information, until this study was directed on this grammar in its final form.

## 5 Findings

### 5.1 The results

The investigation came to the following results: The study found that the ruling regime's religious, historical, and political legitimacy, as well as its relationship with the Hashemites, the nature of Jordanian society, as well as the rule of law and institutions, national unity, and the intimate, mutual relationship between the leadership and the people, were all factors that contributed to Jordan's political stability over the past century. According to the study, the stages of political development that the Jordanian state went through, from the foundation stage to the stage of independence to the stage of participation in government, proceeded at a steady pace with no significant obstacles due to the strength of the relationship between Jordanians on the one hand and Jordanians and the leadership on the other, which is based on positive, acceptance, and sincerity. According to the study, a stable political system is one in which the constitution and the rule of law are respected, a system founded on institutions, the rule of law, and institutions; this is what Jordan has observed throughout its political history since its creation in 1921. The study revealed that Jordan now commands the respect and attention of the entire world as a result of the approach taken by the Jordanian state in its internal policies and external relations, which is based on respect for the law, the rule of the constitution, mutual respect, moderation, honesty, and non-interference in the internal affairs of states except when requesting assistance; this has enabled Jordan to serve as a reference for opinion and advice to be taken in most regional and international forums. According to the study, His Majesty King Abdullah II's focus on the importance of honoring the law and its sovereignty has made all citizens equal before the law, achieving justice and equality. This increases the citizen's attachment to the state and loyalty to the leadership. According to the study, His Majesty King Abdullah II sees Jordan's future as a civil state in which everyone is equal in terms of the rights and duties stipulated in the Jordanian constitution, despite regional developments that have led to Jordan joining the ranks of developed and advanced countries. The study found that the state maintains its national identity and the cohesion of its people with the leadership despite the region's circumstances, which is evidence of the application and activation of the system of regulations and laws, as well as the rule of law for all without exception. According to the study, Jordan has made significant gains in all aspects of education, health, agriculture, industry, trade, and maintaining balanced foreign relations with other countries around the world. According to the study, the Hashemites laid the foundations of Jordan in accordance with the rule of law and institutions, and consolidated the features of freedom, equality, and democracy, and that His Majesty King Abdullah II continues to develop, progress, and reform in all walks of life, and that the Hashemites have a wide, large, and multiple roles in building Jordan, and that Jordan is moving forward in steady steps and beginning its second centennial with strength and confidence.

### 5.2 Results and recommendations discussions

The report proposes the following recommendations based on its findings: Today, everyone is wondering about the secret that helped Jordan to overcome problems, as well as its fate and future in the midst of this upheaval and political conflict. Attempts by some to undermine its security and stability have failed, thanks to the wisdom and tolerance of its Hashemite leadership, Jordanians' faith in their homeland, and the strength and resilience of its security services and armed forces; maintaining it requires everyone to work hard. Jordan, despite the harsh conditions surrounding it, has remained politically and security strong, despite economic challenges that have affected citizens' lives and led to high rates of poverty and unemployment, a high budget deficit and public debt, and economic challenges that are mostly beyond the state's will, such as Syrian asylum, the closure of export borders, the decline in Arab and international aid, the disruption of Egyptian gas, the global financial crisis, and others; this necessitates working to strengthen the home front and finding innovative and swift solutions to all of Jordan's current issues. Those difficult and sometimes unexpected circumstances that coincided created difficult economic conditions and hampered Jordan's economic and development plans from achieving their goals; this necessitates working to find economic and urgent solutions by encouraging foreign investment and providing attractive facilities for it. Jordan's strength and coherence stem from the fact that it is a state of institutions and law. It safeguards and safeguards everyone's rights. It is critical to face any ludicrous activities with force and determination by applying the law and imposing deterrent consequences on transgressors, as well as with justice. Jordan has obstacles as a result of its geopolitical location, including the region's security and political environment. The so-called Deal of the Century or Deal of the Age, which the last US government discussed, and the Zionist media attack on Jordan and its political regime are two of the most visible present political issues. This means maintaining national unity and supporting the Hashemite leadership in confronting all attempts to undermine Jordan's security and stability. There are legitimate concerns that it will have an impact on Jordan's constants and lead to solutions to the Palestinian issue at the expense of Jordan, but everyone must understand that Jordan, led by His Majesty King Abdullah II, will not abandon its national constants, as Jordan will not be an alternative homeland to Palestine. Jordan will not relinquish its rights to the return of all Palestinian refugees and other refugees to their homeland, as well as compensation, in addition to the many water and border issues. His Majesty King Abdullah II's three NOs are clear, and he has told them several times in response to those who challenge Jordan's positions: “no to settlement, no alternative homeland, and no harm to Jerusalem.” Whatever the pressure or price, Jerusalem and its Islamic and Christian sanctuaries are a red line. As a result, it is necessary to confront attempts to tamper with Jordan's social fabric, as well as the discourse of hate and strife promoted by some on social media and other platforms, because Jordanians will not accept neglecting their homeland and its soil, and Jordan is a well-established country with strong leadership, and Jordanians license their blood for the sake of the homeland, and Jordanians believe that the future belongs to them and to Jordan the eternal homeland.

Some believe that deep states can only exist if people are unable to freely organize politically and, as a result, cannot alter their governments through elections or subject military and intelligence services to their established governance [50]. However, this is not the situation in Jordan. Jordan's military was a key player in the region's state creation process. The late King Abdullah I's tribal forces were bolstered by the British-led Arab Legion. Following the succession of his grandson King Hussein to direct command of the force in 1956 as the renamed Royal Jordanian force, it became a major pillar of Jordanian society. As a result of the Jordanian army's composition, no home in Jordan is vacant without a soldier of the Jordanian army or a martyr protecting Jordan. Jordan's deep state is keen to modernize, grow, and arm the Jordanian army with science, knowledge, experience, and ongoing training until it is one of the region's strongest militaries. For example, the Jordanian army's Royal Medical Services are often recognized as the best in the region on humanitarian grounds. Jordanians believe in their army. This is one of the fundamental characteristics that has contributed to Jordan's stability, as well as Jordanians' faith in the military institution, which does not equate to other Jordanians' trust in Jordan.

And others believe that when many Middle Eastern states gained independence, they had a dual state in place, with the formal institutions of the state and a significant shadow state of networks operating behind the scenes. Therefore, many clashes over boundaries, identities, and class privileges continue within Middle Eastern countries because those in positions of power have failed to persuade the public that they have a right to be there [51]. This situation is not in Jordan too; Jordan was founded on a nation-state, as opposed to the other countries in the region, which were built on a national state. Given that Jordan is a republic open to all Arabs, the country's first Prime Minister, Rashid Ṭaliʽa, was Lebanese [52]. Despite its cultural diversity: Jordanian, Arab, Circassian, Chechen, Druze, Kurdish, and Armenian, Jordanian society today represents a national unity that distinguishes Jordan from the rest of the world, in addition to the fact that Christianity and Islam in Jordan serve as a role model rarely seen in the world in terms of love and brotherhood. Jordan was able to pass through difficult stages that ravaged and continue to ravage the region as a result of all of this, relying on Jordanian national unity to enter the second centenary with confidence and stability. Jordanians of all ethnicities and backgrounds collaborated in all sectors to build the Jordanian state without prejudice. Today, Jordan's deep state is working for tripartite reform to carry out political, economic, and administrative modernization, motivated by a belief in the need for the political community to meet its obligations in actively engaging in the construction of the Jordanian state at the start of its bicentennial. To conclude, “real” politics dictate who gets what, when, and how in most of MENA, leaving others to rely on pseudo politics, which is found not only in the deep or shadow states of MENA as some believe, but in every country in the planet.

## 6 Conclusions

Jordan has seen numerous regional crises as a result of Arab-Arab wars, Arab-Israeli tensions, foreign involvement in the Arab area, and Arab Spring events. Jordan is one of five countries that outperform it in terms of human, economic, and military capacities, four of which are Arab countries: Iraq, Syria, Saudi Arabia, and Egypt, with Israel being the fifth. Jordan is located in what has been considered as the world's tensest region for more than a century. Despite this, Jordan was able to maintain its stability and vital role in the region due to the strength of the bond between the loyal Jordanians and their wise Hashemite leadership. Several internal and external factors contributed to internal political stability, including the Hashemite leadership's religious, historical, and political legitimacy, Jordanians' loyalty to the Hashemite leadership, the existence of a constitutional and legal structure and institutions, political moderation and the regime's high degree of political adaptation, and the implementation of gradual political reforms since the state's establishment, and the coherence and professionalism of the military and security institutions, the importance of the political system during crises, the status of social peace, and the increase in citizens' political consciousness. External factors also had a significant role in Jordan achieving internal stability. Jordan has received Western security, political, military, and economic backing (particularly from the United Kingdom and the United States) as well as Arab (particularly from the Gulf States) support from its inception, owing to the importance of Jordan's existence and stability in establishing regional security. However, there are factors that have played and continue to play a role in Jordan's state of political instability at various stages, most notably: Jordan's geographical location in a region of conflicts and disputes, Jordan's organic link to the Palestinian cause and the Arab-Israeli conflict, and Jordan's fear of Israeli and American projects to solve the Palestinian issue on its behalf, the last of which is the so-called Deal of the Century, Arab-Arab dispute, Palestinian asylum, Iraqi asylum, and Syrian asylum, and the security, economic, and social burdens that this entails, the threat of extremism and terrorism from neighboring countries, and Jordan's suffocating economic crisis: budget deficit, high internal and external public debt, and high unemployment, particularly among university graduates and youth, an increase in poverty rates, a decline in citizens' standard of living, the spread of financial and administrative corruption, the preoccupation of Arab countries with their internal problems, and the erosion of Arab solidarity. The study was able to answer the three questions by identifying the factors of political stability in Jordan, the stages of political development in Jordan, and Jordan's position on the world's political map regionally and internationally in the first century of its modern era. The study was also able to test the hypothesis, and the results were identical, indicating that Jordan's stability and legitimacy are based on voluntary consent between the leadership and the people, and on the basis of loyalty to the leadership, belonging to the homeland, the rule of law, and respect for the constitution; this has earned Jordan the world's respect, as the world views Jordan with respect, appreciation, and high credibility in various international forums as a result of Jordanian diplomacy led by King Abdullah II, who represents the voice of reason on Arab and global affairs, has called for the introduction of dialogue on all challenges confronting humanity as a whole.

## Funding statement

The author is grateful to Jadara University, Irbid, Jordan for the financial support granted to cover the publication fee of this research.

## Data Availability Statement

The Data included in article/supp. Material/referenced in article can allow other scholars to reuse these data on the following Links:

Research Gate: https://www.researchgate.net/profile/Mohammad-Bani-Issa-2.

Google Scholar: https://scholar.google.com/citations?view_op=list_works&hl=en&user=GdtGvDgAAAAJ.

Scopus: https://www.scopus.com/authid/detail.uri?authorId=58027695800.

ORCID: https://orcid.org/0000-0002-3179-1613.

LinkedIn: https://www.linkedin.com/in/dr-mohammad-bani-issa-1b338318.

Data is available also at SSRN: https://doi.org/10.2139/ssrn.3936321

## CRediT authorship contribution statement

**Mohammad Saleh Bani Issa:** Conceptualization, Data curation, Formal analysis, Methodology, Project administration, Resources, Writing – original draft, Writing – review & editing.

## Declaration of competing interest

The authors declare that they have no known competing financial interests or personal relationships that could have appeared to influence the work reported in this paper.

## References

[1] (2022). See also, the pottery of Jordan, A manual, edited by jehad haron and douglas R. Clark, American center of research & madaba regional archaeological museum project. https://rhc.jo/ar/jordan-and-jordanians/.

[2] (2023). See also, Jordan ayyubid period (1174 - 1236 A.D), Jordan history last updated: 13 August. https://kindian.com/review/21286/.

[3] (1988). Muneeb Al-Madhi; Al-Mousa, Suleiman, (1988): A History of Jordan in the Twentieth Century.

[4] (2021). Why was the Jordan River called by that name? see also, what is the significance of the Jordan River and why is it called by that name? September. https://mawdoo3.com/.

[5] Ababsa Myriam (2013).

[6] Mahfouz M. (2006).

[7] Rashwani M. (2003).

[8] Eli Margolis J. (2010). Understanding political stability and instability. Journal Civil Wars.

[9] See also. https://kingabdullah.jo/ar/page/the-hashemites.

[10] (1983). Abu Hisham Abdullah; Ben Seddik, Quraish Family's Notables of Mecca Protected. *Jeddah, Saudi Arabia*.

[11] Schlumberger O., Bank A. (2002). Succession, legitimacy, and regime stability in Jordan. The Arab Studies Journal.

[12] Hashemite. https://emirate.wiki/wiki/Sharif.%20See%20also.

[13] Nassuri Ahmed (2008). The political system and the dialectic of legitimacy and legitimacy. Damascus University Journal of Economic and Legal Sciences.

[14] The great arab-revolution. See also, Jordan history, the great Arab revolt. https://ebook.univeyes.com/68978/pdf.

[15] Correspondence, Hussein-McMahon. See also, Jordan history, the clash of promises and interests. https://www.marefa.org/.

[16] Gubser Peter (1991).

[17] Schlumberger O., Bank A. (2002). Succession, legitimacy, and regime stability in Jordan. The Arab Studies Journal.

[18] (2020). The Papers of Al-Sharif Hussein Bin Ali, Documents Published Together for the First Time, Prepared by Mohammad Yunes Al-Abbadi Translated by Lamya Al-Khraisha.

[19] (1998). The Hashemites Laid the Foundations of the Jordanian State in Accordance with the Rule of Law and Institutions. See Also, “The Hashemite Option” Chapter 10, in the Shadow of the Prophet Milton Viorst.

[20] (2012). Developing political life has been the first concern of our Hashemite leadership since the inception of the emirate, 28/5. https://www.almadenahnews.com/article/152784.

[21] (2012). Fawaz Abdul-Rahman and Others, the Jordanian Society, Al-Manhal, Amman.

[22] Jordanian society/national and civic education. https://joacademy.com/eschool/index/lesson/3361.

[23] (2018). Waheeb Al-Shaer, Formation of Jordanian Society, Amman.

[24] (2021). See also, the historical encyclopedia of the Jordan armed forces- Arab army history of bravery and sacrifices. https://fanack.com/ar/jordan/population-of-jordan/.

[25] Eilon Joab B., Alon Yoav (2007).

[26] Hamarneh Mustafa (2005).

[27] Al-Dajani Munther (1993).

[28] Mohafazah Ali (1990).

[29] Ababsa Myriam (2013). ATLAS of JORDAN: the Hashemites and the Creation of Transjordan.

[30] Ababsa Myriam (2013). ATLAS of JORDAN: the Hashemites and the Creation of Transjordan.

[31] The evolution of political life in Jordan. 24 May (2015), see also. http://alrai.com/article/715756.html.

[32] Rudd Jeffery A. (1993).

[33] Bentwich Norman (1929). The mandate for transjordan. British yearbook of international law. Humphrey Sumner Milford.

[34] King Hussein's diary. See also, Uneasy Lies The Head, A king speaks out. http://Http://r.wikipedia.org/wiki.

[35] Al-Adwan Mustafa (2001).

[36] Al-Bataineh Rafea (2004).

[37] Satloff R., Schener D. (2015).

[38] (2010). Government policies and indicators of political and economic stability, an analytical study (2001-2010). Journal of the Union of Arab Universities for Literature.

[39] Tarawneh Raafat (2004).

[40] (2018). Royal speeches. See also, youth WELL-BEING policy review of Jordan © oecd. https://kingabdullah.jo/.

[41] Mashaqbeh Amin (1989).

[42] Khdeirat O. (1990). The middle class and its impact on political stability in Jordan. Journal of Arab and International Studies.

[43] Mashaqbeh Amin (1989).

[44] Bani Hamad A.H. (2019). The impact of political reforms on stability in Jordan: (2011-2018). Open J. Polit. Sci..

[45] Vatikiotis P.J. (2017).

[46] See also, Jordan human resources, the demographic challenge. https://www.raialyoum.com/85.

[47] (2019). /2620470, see also, Water Policy.

[48] (2016). HUSSAM HUSSEIN, an Analysis of the Discourse of Water Scarcity and Hydro Political Dynamics in the Case of Jordan.

[49] (2020). See also, Two-state solution ‘crystal clear’ path to peace — fm. https://www.mfa.gov.jo/content/85.

[50] For more information on the deep state, please visit this website: springborg, Robert; Deep States in MENA. https://mepc.org/journal/deep-states-mena.

[51] For more information on the shadow state, please visit this website: charles Tripp; the fragility of Middle Eastern States. https://www.joinexpeditions.com/exps/1080.

[52] For further information about Jordanian prime ministers. See also. https://en.wikipedia.org/wiki/List_of_prime_ministers_of_Jordan.

